# Value of turbo spin echo–based diffusion-weighted imaging in the differential diagnosis of benign and malignant solitary pulmonary lesions

**DOI:** 10.1038/s41598-024-60423-w

**Published:** 2024-04-30

**Authors:** Qiang Lei, Lishan Liu, Jianneng Li, Kanghui Yu, Yi Yin, Jurong Wang, Sulian Su, Yang Song, Guihua Jiang

**Affiliations:** 1 Department of Medical Imaging, Guangdong Second Provincial General Hospital, Shiliugang Road, Haizhu District, Guangzhou, 510317 People’s Republic of China; 2 Department of Radiology, The Fifth Affiliated Hospital of Guangzhou Medical University, 621 Gangwan Road, Guangzhou, 510799 People’s Republic of China; 3 Department of Radiology, Xiamen Humanity Hospital Fujian Medical University, Xianyue Road, Huli District, Xiamen, 361000 People’s Republic of China; 4 MR Scientific Marketing, Siemens Healthineers Ltd., 399 Haiyang West Road, Pudong New Area, Shanghai, 200126 People’s Republic of China

**Keywords:** Lung neoplasms, Diffusion-weighted magnetic resonance imaging, Area under the curve, Sensitivity and specificity, Cancer, Computational biology and bioinformatics, Diseases, Medical research, Oncology

## Abstract

To quantitatively assess the diagnostic efficacy of multiple parameters derived from multi-*b*-value diffusion-weighted imaging (DWI) using turbo spin echo (TSE)–based acquisition techniques in patients with solitary pulmonary lesions (SPLs). A total of 105 patients with SPLs underwent lung DWI using single-shot TSE–based acquisition techniques and multiple *b* values. The apparent diffusion coefficient (ADC), intravoxel incoherent motion (IVIM) parameters, and lesion-to-spinal cord signal intensity ratio (LSR), were analyzed to compare the benign and malignant groups using the Mann–Whitney U test and receiver operating characteristic analysis. The D_star_ values observed in lung cancer were slightly lower than those observed in pulmonary benign lesions (28.164 ± 31.950 versus 32.917 ± 34.184; Z = -2.239, *p* = 0.025). The LSR values were significantly higher in lung cancer than in benign lesions (1.137 ± 0.581 versus 0.614 ± 0.442; Z = − 4.522, *p* < 0.001). Additionally, the ADC_800_, ADC_total_, and D values were all significantly lower in lung cancer than in the benign lesions (Z = − 5.054, -5.370, and -6.047, respectively, all *p* < 0.001), whereas the f values did not exhibit any statistically significant difference between the two groups. D had the highest area under the curve (AUC = 0.887), followed by ADC_total_ (AUC = 0.844), ADC_800_ (AUC = 0.824), and LSR (AUC = 0.789). The LSR, ADC_800_, ADC_total_, and D values did not differ statistically significantly in diagnostic effectiveness. Lung DWI using TSE is feasible for differentiating SPLs. The LSR method, conventional DWI, and IVIM have comparable diagnostic efficacy for assessing SPLs.

## Introduction

Lung cancer continues to be one of the most common malignancies worldwide and is the leading cause of cancer-related deaths^1^. Diagnosing and characterizing solitary pulmonary lesions (SPLs), ranging from lung cancer to infections and other benign tumors, are still a major medical challenge^2^. Magnetic resonance imaging (MRI) is emerging as an alternative and complementary to lung imaging^3^. Lung MRI is more advantageous than chest radiography and computed tomography (CT) in terms of avoiding ionizing radiation. Moreover, diffusion weighted imaging (DWI) has the potential to gather diffusion and perfusion data, aiding in the characterization of SPLs. However, addressing geometric distortion resulting from magnetic field inhomogeneities in lung MRI is crucial. Therefore, effective methods should be developed to obtain high-quality images while simultaneously ensuring the precision of the derived parameters.

DWI is a noninvasive modality used to assess the diffusivity of water molecules within living tissues. Intravoxel incoherent motion DWI (IVIM-DWI), which uses a biexponential model, enables the separate determination of diffusion and perfusion parameters, unlike mono-exponential DWI^4^. Echo-planar imaging (EPI) is the preferred DWI technique because of its rapid acquisition time^5^. Also, many studies^6,7^ including meta-analysis studies^8,9^ have demonstrated the considerable benefit of EPI-DWI for the diagnosis and characterization of pulmonary nodules. However, the presence of magnetic field inhomogeneities in the lungs can lead to signal loss or geometric distortion in pulmonary nodule EPI-DWI, thereby compromising the accuracy of pulmonary nodule measurements of derived parameters.

Turbo spin echo–based DWI may be a useful alternative to EPI-DWI in the lung, which is particularly susceptible to magnetic inhomogeneity. Turbo spin echo (TSE) is the most frequently used DWI technique less sensitive to magnetic inhomogeneity. Previously published studies^10–12^ have highlighted the ability of TSE-DWI to improve image quality in conditions such as head and neck cancers, orofacial lesions, and breast cancer. In addition, a recent study^13^ already presented that TSE-DWI provides significantly improved image quality and test–retest robustness of IVIM parameters in patients with SPLs. However, its role in the assessment of pulmonary lesions remains to be elucidated. As part of further validating lung TSE-DWI feasibility, it should be valuable to explore and compare the role of TSE-DWI in the diagnosis and characterization of SPLs.

The objective of this study was to explore and compare the diagnostic efficacy of the lesion-to-spinal cord signal intensity ratio (LSR) method, diffusion, and perfusion parameters derived from multi-*b*-value TSE-DWI in patients with SPLs. Furthermore, to the best of our knowledge, this study represents the initial utilization of multi-*b*-value TSE-DWI and its demonstration of diagnostic efficacy in lung cancer. We believe that the proposed method is a highly promising alternative to lung diffusion-weighted magnetic resonance imaging currently used in clinical practice.

## Materials and methods

### Patient population

**Figure 1:**
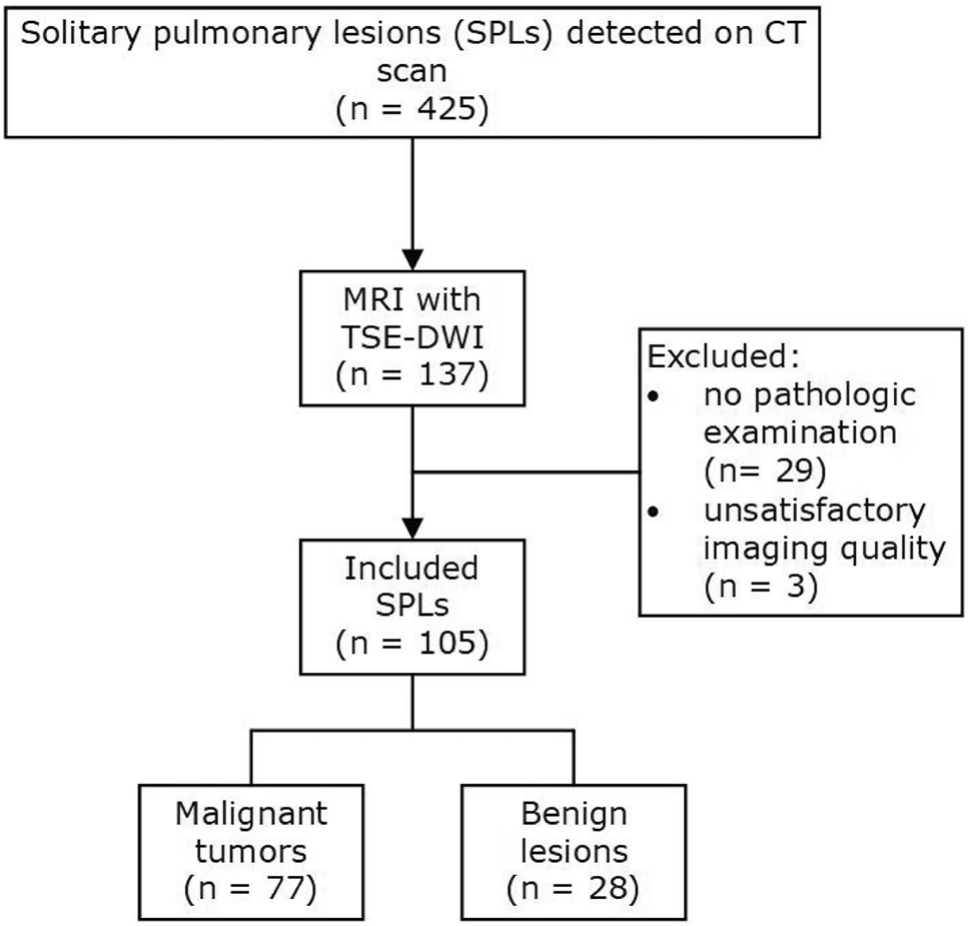
Flow diagram of the study. Note- DWI = diffusion weighted imaging; TSE = turbo spin echo.

This study was approved by the institutional review board of Guangdong Second Provincial General Hospital (protocol number 2023-DW-KZ-060–02), and written informed consent was obtained from all enrollees. Patients with solitary pulmonary lesions detected by chest CT at our institution from March 2018 to August 2023. The inclusion criteria were as follows: (a) a maximum tumor diameter of more than 10 mm as measured on chest CT images, and with ground glass, necrosis, calcification, and cavity components comprising less than one-third of the lesion; (b) no contraindication for MRI examinations; and (c) patients who had not undergone any treatments. A total of 105 patients (66 males and 39 females; age range, 19–78 years; mean age, 54 years) diagnosed with SPLs through chest CT examination, including 77 malignant tumors (47 invasive adenocarcinomas, 15 squamous carcinomas, six small-cell carcinomas, five lymphoid epithelioid carcinomas, two mucosa-associated lymphoid tissue lymphomas, one carcinoid, and one mucoepidermoid carcinoma) and 28 benign lesions (two hamartomas, five tuberculous granuloma, five cryptococcal granuloma, and 16 chronic organizing inflammation) were enrolled in our study after excluding 32 patients according to exclusion criteria (Fig. 1). The pathological information was obtained or planned by surgical resection or transbronchial or transthoracic biopsy, and the interval between the lung DWI and biopsy was less than 30 days.

### Image acquisition and postprocessing

With the use of a 3.0 T MRI scanner equipped with a 16-channel body phased array coil (Achieva; Philips Healthcare, Best, the Netherlands), axial T1-weighted imaging, T2-weighted imaging, and DWI were performed for all examinations. DWI was acquired during free breathing using a single-shot TSE–based sequence (Table 1).

**Table 1:** Scanning parameters of magnetic resonance imaging.

	T1WI	T2WI	TSE-DWI
TR (ms)	10	937	1000
TE (ms)	2.3	80	60
NSA	1	1	2
FOV (mm)	430 × 342	430 × 349	260 × 423
Slice thickness (mm)	5	7	5
Gap (mm)	0.5	0.7	0
*b* value (s/mm^2^)	–	–	0, 20, 40, 200, and 800
Directions	–	–	3
Matrix	288 × 171	360 × 247	88 × 140
Scanning time	47 s	23 s	4min16s

*TE* echo time, *TR* repetition time, *NSA* number of signals acquired, *FOV* field of view, *m* minutes, *s* seconds.

DWI data were postprocessed using PRIDE software (Philips Healthcare) to generate apparent diffusion coefficient (ADC) maps with *b* values of 0 and 800 s/mm^2^. In IMAge/enGINE MRI Diffusion Toolbox (Beta V2.0.3; Vusion Tech, Hefei, China) with monoexponential and biexponential fit models, ADC_total_ and IVIM parameters were calculated, respectively, for all *b* values from 0 to 800 s/mm^2^.

The ADC value was calculated based on the following equation:

$$ S\left( b \right)/S_{0} \, = \,{\text{exp}}( - \,b{\text{ADC}}) $$

According to the biexponential IVIM model, the correlation between DWI signal intensity (SI) and the *b*-factor is as follows^4^:

$$ S\left( b \right)/{\text{S}}_{0} \, = \,{\text{f}}.{\text{exp}}( - \,b{\text{D}}_{{{\text{star}}}} )\, + \,\left( {{\text{1}}\, - \,{\text{f}}} \right).{\text{exp}}( - \,b{\text{D}})  $$

where *S*_0_ and *S*(*b*) are the diffusion-weighted signal intensities for *b* = 0 s/mm^2^ and a preset *b* value, respectively.

All regions of interest (ROIs) were manually drawn in the solid lesion on the slice with maximum transverse diameter, avoiding necrosis and hemorrhage, as well as the spinal cord (12–16 mm^2^) for mean SI measurements, under the guidance of an experienced radiologist. LSR was calculated using the following equation^14^:

$$  {\text{LSR}}\, = \,{\text{SI}}_{{{\text{lesion}}}} /{\text{SI}}_{{{\text{spinal cord}}}}    $$

### Statistical analysis

Data that followed a normal distribution were reported as mean (standard deviation), and data that followed a non-normal distribution were reported as median (interquartile range). With the use of SPSS (version 22.0, IBM Corporation, USA) and MedCalc software (version 18.2.1, Mariakerke, Belgium), the Mann–Whitney U test and receiver operating characteristic analysis were performed. A *p* value less than 0.05 was considered statistically significant.

### Ethics approval

All procedures performed in studies involving human participants were in accordance with the ethical standards of the institutional and/or national research committee and with the 1964 Helsinki declaration and its later amendments or comparable ethical standards.

### Informed consent

This retrospective study was approved by the medical ethical committee of Guangdong Second Provincial General Hospital, and informed written consent was obtained from all enrollees.

## Results

### MRI appearance

**Figure 2:**
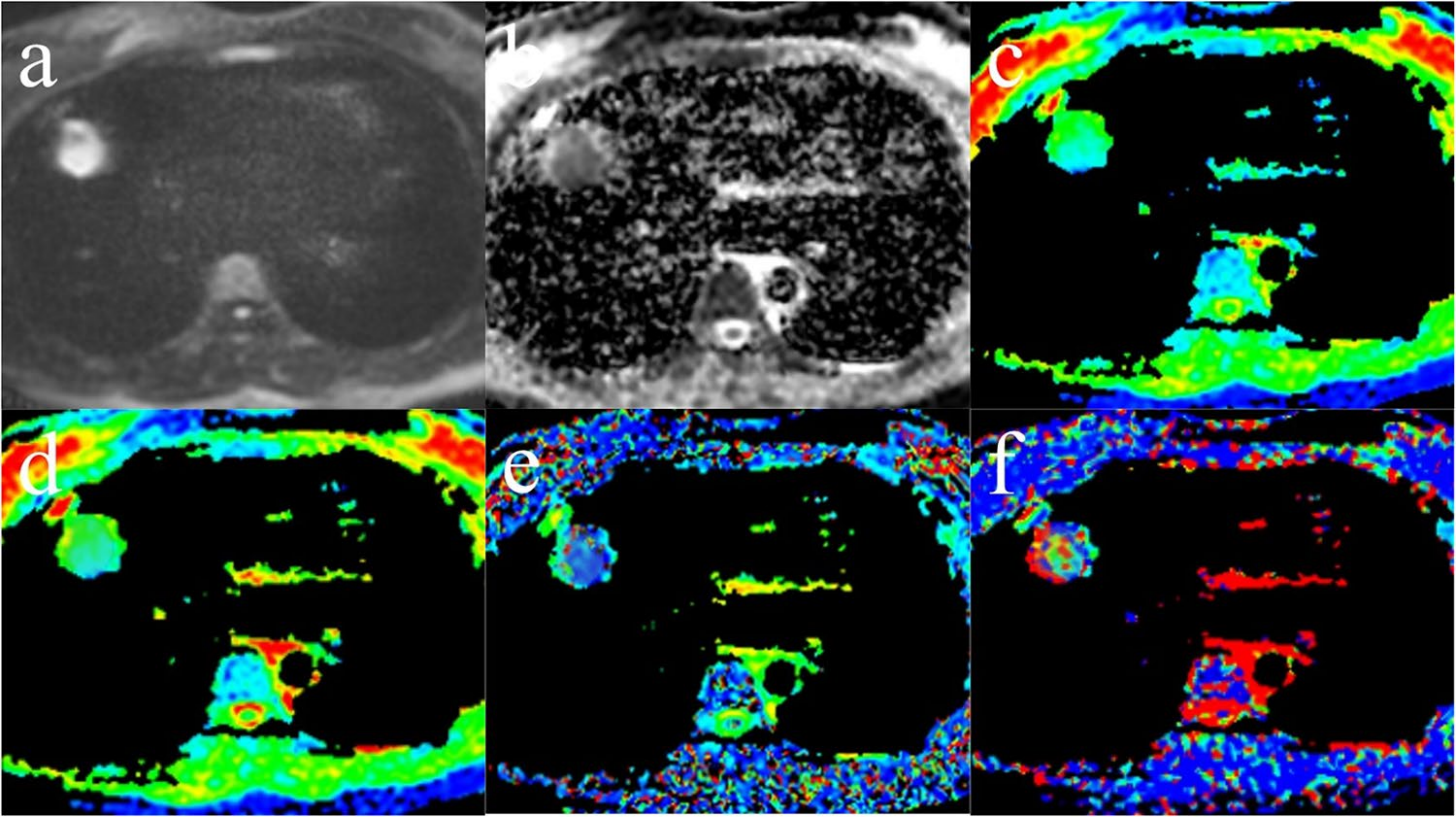
Pulmonary invasive adenocarcinoma in the right middle lobe. (**a**) Diffusion-weighted imaging (DWI) (*b* = 800 s/mm^2^); the signal intensity (SI) of the lesion was increased, and the lesion-spinal signal intensity ratio (LSR) was 1.230. (**b**) ADC_800_ map. (**c**) ADC_total_ map. (**d**) Pure diffusion coefficient (**D**) map. (e) Perfusion fraction (f) map. (f) Perfusion-related diffusion coefficient (D_star_) map.

**Figure 3:**
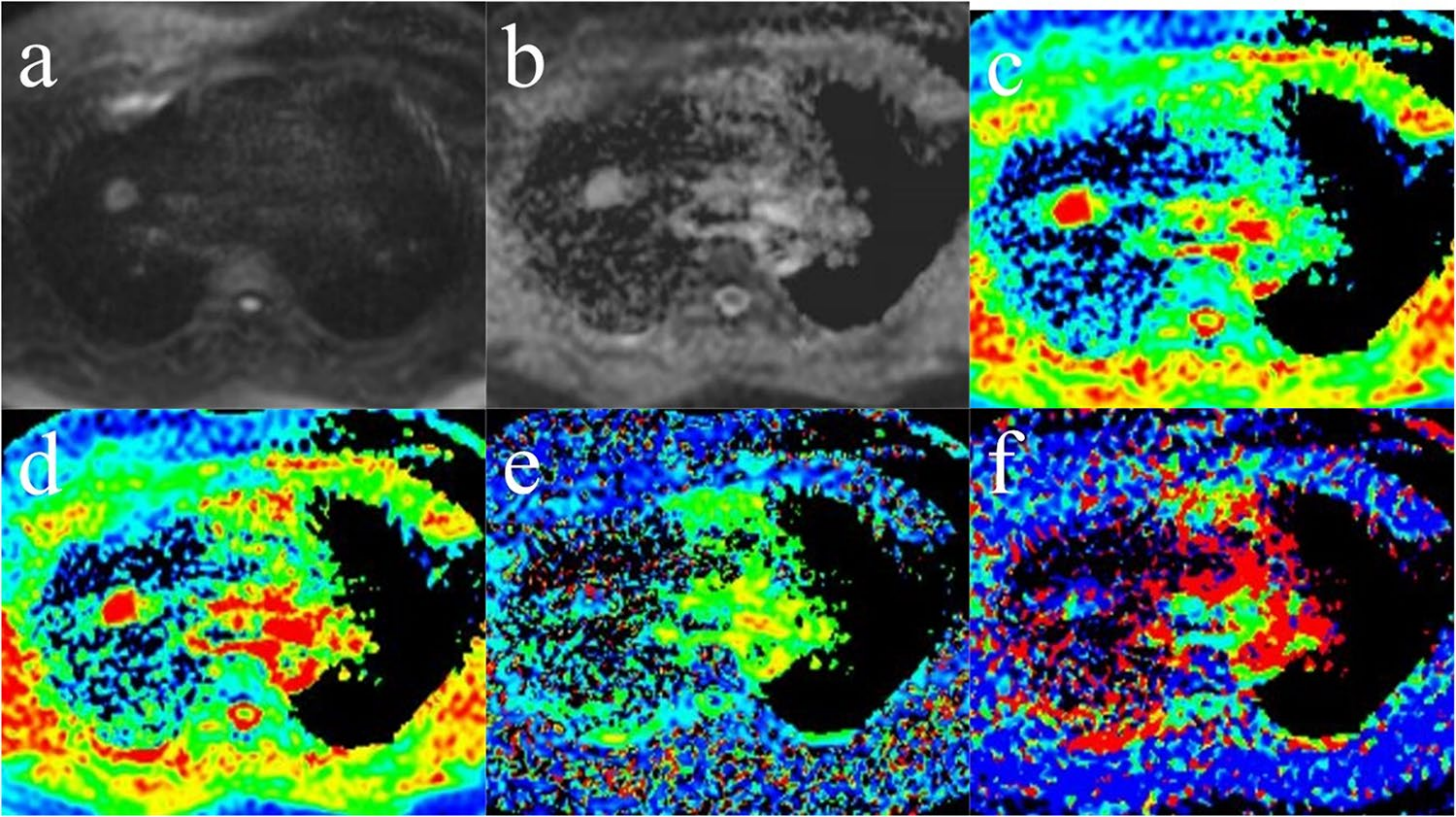
Pulmonary hamartoma in the right upper lobe. (**a**) Diffusion-weighted imaging (DWI) (*b* = 800 s/mm^2^); the signal intensity (SI) of the lesion was decreased, and the lesion-spinal signal intensity ratio (LSR) was 0.530. (**b**) ADC_800_ map. (**c**) ADC_total_ map. (**d**) Pure diffusion coefficient (**D**) map. (**e**) Perfusion fraction (**f**) map. (**f**) Perfusion-related diffusion coefficient (D_star_) map.

The maximum diameters of the SPLs at the largest level ranged from 1.000 to 9.800 cm, with a mean of 1.700 cm. Figures 2 and 3 depict the representative multi-*b*-value TSE-IVIM-DWI appearance of two SPLs pathologically confirmed to be benign and malignant.

### Diagnostic performance of multiple parameters

Table 2 presents the LSR, ADC_800_, ADC_total_, D, f, and D_star_ values for both lung cancer and benign groups.

**Table 2:** Diffusion parameters of malignant and benign solitary pulmonary lesions.

Parameters	Benign	Malignant	Z	*P*
LSR	0.614(0.442)	1.137(0.581)	− 4.522	0.000
ADC_800_	1.375(0.439)	1.155(0.240)	− 5.054	0.000
ADC_total_	1.247(0.412)	1.025(0.256)	− 5.370	0.000
D	1.470(0.511)	1.077(0.265)	− 6.047	0.000
f	0.300(0.127)	0.254(0.150)	− 1.583	0.113
D_star_	32.917(34.184)	28.164(31.950)	− 2.239	0.025

ADC, D, and D_star_ are in units of × 10^–3^ mm^2^/s.

Data as median (IQR), Mann–Whitney U test, *ADC* apparent diffusion coefficient, *D* pure diffusion coefficient, *f* perfusion fraction, *D*_*star*_ perfusion-related diffusion coefficient, *LSR* lesion-spinal signal intensity ratio.

**Figure 4:**
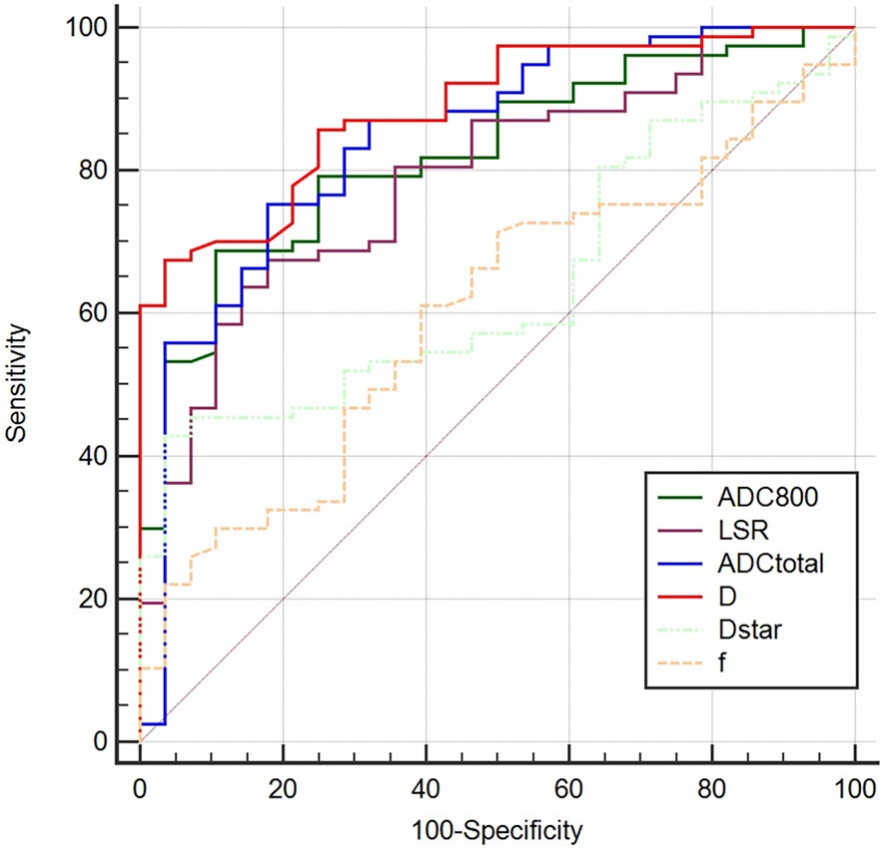
Results of receiver operating characteristic analysis for lesion-spinal signal intensity ratio (LSR), ADC_800_, ADC_total_, pure diffusion coefficient (**D**), perfusion fraction (**f**), and perfusion-related diffusion coefficient (D_star_) derived from TSE-DWI in differentiating solitary pulmonary lesions.

Figure 4 shows the receiver operating characteristic curves used to predict lung cancer. The AUC values for LSR and ADC_800_ were determined to be 0.789 and 0.824, respectively; the corresponding cutoff value, sensitivity, and specificity were found to be 0.954, 67.5%, and 82.1%, respectively, for LSR and 1.268, 68.8%, and 89.3%, respectively, for ADC_800_. In addition, the AUC values for ADC_total_ and D were 0.844 and 0.887, respectively; the corresponding cutoff value, sensitivity, and specificity were 1.131, 75.3%, and 82.1%, respectively, for ADC_total_ and 1.182, 67.5%, and 96.4%, respectively, for D (Table 3). However, there were no significant differences in diagnostic efficacy between LSR, ADC_800_, ADC_total_, and D, as presented earlier.

**Table 3:** Sensitivity and specificity of diffusion parameters at optimal cutoff values in differentiating malignant from benign solitary pulmonary lesions.

Parameters	AUC	Cutoff value	Sensitivity(%)	Specificity(%)	+ LR(%)	−LR(%)
LSR	0.789	0.954	67.5	82.1	3.8	0.4
ADC_800_	0.824	1.268	68.8	89.3	6.4	0.4
ADC_total_	0.844	1.131	75.3	82.1	4.2	0.3
D	0.887	1.182	67.5	96.4	18.9	0.3
D_star_	0.643	20.118	42.9	96.4	12.0	0.6

*AUC* area under the ROC curve, *ADC* apparent diffusion coefficient, *D* pure diffusion coefficient, *D*_*sta*r_ perfusion-related diffusion coefficient, *LSR* lesion-to-spinal cord signal intensity ratio, + *LR* positive likelihood ratio, − *LR* negative likelihood ratio.

## Discussion

Lung cancer remains a major threat to human health. The results showed that the TSE technique is considerable clinical promise as an alternative to diffusion-weighted magnetic resonance imaging of the lung. Furthermore, the findings of this study demonstrated that multi-*b*-value TSE-DWI is both clinically feasible and valuable in distinguishing between malignant and benign SPLs. The results also showed that lung cancer had notably lower values of diffusion-related parameters D and ADC, as well as a higher LSR value, than did benign lesions. This difference can be attributed to the high cell density of malignant tumor tissues, which results in increased brightness on DWI due to the restricted diffusion.

This study was performed using TSE-based acquisition techniques, in contrast to most existing lung DWI studies. EPI is still the clinical mainstream technique for DWI because of its extensive usage^3^. Although echo-planar acquisition has the advantage of reducing the acquisition time for DWI, it still presents significant limitations, especially for some specific site applications, such as lung imaging. These limitations include susceptibility to motion artifacts and geometric distortion caused by magnetic field inhomogeneity, which are more prominent in precise lung DWI. This is primarily attributed to the accumulation of phase errors in echo planar–based acquisition, which can result in signal loss or image distortion. There are some ways to reduce geometric distortion, such as reverse phase encoding to correct EPI-related distortion^15^ and replacing EPI with TSE^16^. Diffusion-weighted TSE is less sensitive to magnetic inhomogeneity existing at the air–lung interface and exhibits better geometrical accuracy than EPI-DWI^17^.

Our study found that diffusion-related parameters derived from TSE-IVIM-DWI, D, and ADC values could distinguish between malignant and benign SPLs and possess higher diagnostic accuracy than D_star_ and f, consistent with most lung DWI studies using echo planar–based acquisition techniques^18,19^. Furthermore, the AUC of D was slightly higher than that of ADC_total_, followed by ADC_800_. Also, D had the highest specificity, and ADC achieved the highest sensitivity, in line with some studies^6^, but not with others^18^. These inconsistent results may be caused by the bias of the included cases and lesion size and variance in acquisition techniques. Nevertheless, these findings suggest that combining D and ADC in clinical practice could be more accurate than using either index alone.

IVIM perfusion-related D_star_ and f values did not demonstrate a good diagnostic efficacy for differentiating malignant SPLs from benign SPLs, consistent with most previous studies^6,18,19^, but not the same as the studies conducted by Deng et al.^20^ and Wang et al.^21^. The inconsistencies between these studies may be associated with the diverse pathological types included in each study and the poor measurement reproducibility of D_star_ and f. Ultimately, D_star_ and f values could be used to obtain tissue perfusion information without the injection of contrast agents, and this possibility should be confirmed in future studies.

In our study, we found that the LSR method can be valuable in distinguishing malignant SPLs from benign SPLs. The LSR values exhibit a notable increase in lung cancer cases compared with the benign group, aligning with previous research findings^14,22^. The LSR method demonstrates satisfactory diagnostic precision and presents itself as a practical and efficacious alternative for regular clinical implementation. However, the reproducibility of the LSR method may be a challenge, as it does rely on subjective evaluation.

This study has several limitations. First, echo planar–based acquisition, the most common DWI technique, was not performed as a control group, which may have resulted in excessive scan times. A small preliminary pilot study by our group indicated no statistically significant difference in the diagnostic efficacy of EPI-DWI and TSE-DWI^16^. Second, only nodules larger than 10 mm in diameter and pathologically confirmed were included; including all nodules encountered in clinical practice may yield different results. In addition, the analysis solely encompassed a comparison between lung cancer and benign SPL groups and did not consider the classification of benign lesions as benign tumors or inflammatory granulomas. Furthermore, additional investigation is necessary to explore various pathological types and degrees of differentiation in malignant lesions.

In conclusion, lung DWI using TSE-based acquisition techniques is feasible for differentiating malignant SPLs from benign SPLs. Both the LSR method and conventional DWI perform similarly to IVIM in the assessment of SPLs.

## Data Availability

All the materials and data in studies are available. Qiang Lei should be contacted if someone wants to request the data from this study.

## References

[1] Sung H, Ferlay J, Siegel RL (2021). Global Cancer Statistics 2020: GLOBOCAN estimates of incidence and mortality worldwide for 36 cancers in 185 countries. CA Cancer J. Clin..

[2] Thai AA, Solomon BJ, Sequist LV, Gainor JF, Heist RS (2021). Lung cancer. Lancet.

[3] Sim AJ, Kaza E, Singer L, Rosenberg SA (2020). A review of the role of MRI in diagnosis and treatment of early stage lung cancer. Clin. Transl. Radiat. Oncol..

[4] Le Bihan D, Breton E, Lallemand D, Grenier P, Cabanis E, Laval-Jeantet M (1986). MR imaging of intravoxel incoherent motions: Application to diffusion and perfusion in neurologic disorders. Radiology.

[5] Ahlawat S, Fayad LM (2018). Diffusion weighted imaging demystified: The technique and potential clinical applications for soft tissue imaging. Skelet. Radiol.

[6] Jiang J, Fu Y, Hu X (2020). The value of diffusion-weighted imaging based on monoexponential and biexponential models for the diagnosis of benign and malignant lung nodules and masses. Br. J. Radiol..

[7] Zhu Q, Ren C, Xu JJ, Li MJ, Yuan HS, Wang XH (2021). Whole-lesion histogram analysis of mono-exponential and bi-exponential diffusion-weighted imaging in differentiating lung cancer from benign pulmonary lesions using 3 T MRI. Clin. Radiol..

[8] Chen Y, Han Q, Huang Z (2022). Value of IVIM in differential diagnoses between benign and malignant solitary lung nodules and masses: A meta-analysis. Front. Surg..

[9] Liang J, Li J, Li Z (2020). Differentiating the lung lesions using Intravoxel incoherent motion diffusion-weighted imaging: A meta-analysis. BMC Cancer.

[10] Mikayama R, Yabuuchi H, Sonoda S (2018). Comparison of intravoxel incoherent motion diffusion-weighted imaging between turbo spin-echo and echo-planar imaging of the head and neck. Eur. Radiol..

[11] Mori N, Mugikura S, Miyashita M (2021). Turbo spin-echo diffusion-weighted imaging compared with single-shot echo-planar diffusion-weighted imaging: Image quality and diagnostic performance when differentiating between ductal carcinoma in situ and invasive ductal carcinoma. Magn. Reson. Med. Sci..

[12] Panyarak W, Chikui T, Yamashita Y, Kamitani T, Yoshiura K (2019). Image quality and ADC assessment in turbo spin-echo and echo-planar diffusion-weighted MR imaging of Tumors of the head and neck. Acad. Radiol..

[13] Wan Q, Lei Q, Wang P (2020). Intravoxel incoherent motion diffusion-weighted imaging of lung cancer: Comparison between turbo spin-echo and echo-planar Imaging. J. Comput. Assist. Tomogr..

[14] Uto T, Takehara Y, Nakamura Y (2009). Higher sensitivity and specificity for diffusion-weighted imaging of malignant lung lesions without apparent diffusion coefficient quantification. Radiology.

[15] Colletti PM (2020). Reverse phase encoding-corrected DWI improves MRI for PET/MRI of lung cancer. Radiology.

[16] Lei Q, Wan Q, Liu L (2021). Values of apparent diffusion coefficient and lesion-to-spinal cord signal intensity in diagnosing solitary pulmonary lesions: Turbo spin-echo versus echo-planar imaging diffusion-weighted imaging. Biomed. Res. Int..

[17] Tyagi N, Cloutier M, Zakian K, Deasy JO, Hunt M, Rimner A (2019). Diffusion-weighted MRI of the lung at 3T evaluated using echo-planar-based and single-shot turbo spin-echo-based acquisition techniques for radiotherapy applications. J. Appl. Clin. Med. Phys..

[18] Wan Q, Deng YS, Lei Q (2019). Differentiating between malignant and benign solid solitary pulmonary lesions: Are intravoxel incoherent motion and diffusion kurtosis imaging superior to conventional diffusion-weighted imaging?. Eur. Radiol..

[19] Kumar N, Sharma M, Aggarwal N (2021). Role of various DW MRI and DCE MRI parameters as predictors of malignancy in solid pulmonary lesions. Can. Assoc. Radiol. J..

[20] Deng Y, Li X, Lei Y, Liang C, Liu Z (2016). Use of diffusion-weighted magnetic resonance imaging to distinguish between lung cancer and focal inflammatory lesions: A comparison of intravoxel incoherent motion derived parameters and apparent diffusion coefficient. Acta Radiol..

[21] Wang LL, Lin J, Liu K (2014). Intravoxel incoherent motion diffusion-weighted MR imaging in differentiation of lung cancer from obstructive lung consolidation: Comparison and correlation with pharmacokinetic analysis from dynamic contrast-enhanced MR imaging. Eur. Radiol..

[22] Henz Concatto N, Watte G, Marchiori E (2016). Magnetic resonance imaging of pulmonary nodules: Accuracy in a granulomatous disease-endemic region. Eur. Radiol..

